# Uncovering the Achilles heel of genetic heterogeneity: machine learning-based classification and immunological properties of necroptosis clusters in Alzheimer’s disease

**DOI:** 10.3389/fnagi.2023.1249682

**Published:** 2023-09-20

**Authors:** Huangwei Wei, Chunle Wu, Yulin Yuan, Lichuan Lai

**Affiliations:** ^1^Department of Neurology, The People’s Hospital of Guangxi Zhuang Autonomous Region, Nanning, China; ^2^Department of Blood Transfusion, The People’s Hospital of Guangxi Zhuang Autonomous Region, Nanning, China; ^3^Department of Laboratory, The People’s Hospital of Guangxi Zhuang Autonomous Region, Nanning, China

**Keywords:** Alzheimer’s disease, necroptosis, immune landscapes, diagnostic model, machine learning algorithm

## Abstract

**Background:**

Alzheimer’s disease (AD) is an age-associated neurodegenerative disease, and the currently available diagnostic modalities and therapeutic agents are unsatisfactory due to its high clinical heterogeneity. Necroptosis is a common type of programmed cell death that has been shown to be activated in AD.

**Methods:**

In this study, we first investigated the expression profiles of necroptosis-related genes (NRGs) and the immune landscape of AD based on GSE33000 dataset. Next, the AD samples in the GSE33000 dataset were extracted and subjected to consensus clustering based upon the differentially expressed NRGs. Key genes associated with necroptosis clusters were identified using Weighted Gene Co-Expression Network Analysis (WGCNA) algorithm, and then intersected with the key gene related to AD. Finally, we developed a diagnostic model for AD by comparing four different machine learning approaches. The discrimination performance and clinical relevance of the diagnostic model were assessed using various evaluation metrics, including the nomogram, calibration plot, decision curve analysis (DCA), and independent validation datasets.

**Results:**

Aberrant expression patterns of NRGs and specific immune landscape were identified in the AD samples. Consensus clustering revealed that patients in the GSE33000 dataset could be classified into two necroptosis clusters, each with distinct immune landscapes and enriched pathways. The Extreme Gradient Boosting (XGB) was found to be the most optimal diagnostic model for the AD based on the predictive ability and reliability of the models constructed by four machine learning approaches. The five most important variables, including ACAA2, BHLHB4, CACNA2D3, NRN1, and TAC1, were used to construct a five-gene diagnostic model. The constructed nomogram, calibration plot, DCA, and external independent validation datasets exhibited outstanding diagnostic performance for AD and were closely related with the pathologic hallmarks of AD.

**Conclusion:**

This work presents a novel diagnostic model that may serve as a framework to study disease heterogeneity and provide a plausible mechanism underlying neuronal loss in AD.

## Introduction

Alzheimer’s disease (AD) is a devastating age-associated neurodegenerative disease, mainly characterized by progressive deterioration of memory and cognitive function (Huynh et al., 2020). According to the latest statistics, China has 15 million cases aged 60 years with dementia, including 9.83 million with AD, and new cases are increasing being reported at an alarming rate (Ren et al., 2022). Epidemiological status of AD in the United States and European nations also present a similar phenomenon, with researchers predicting that there will be 20 million cases of AD in the United States by 2050 (Richard and Mousa, 2022). In this context, the enduring adverse effects on the quality of life and the associated comorbidities experienced by patients impose a significant burden on economies and healthcare systems globally (Jia et al., 2018). Latest scientific developments have expanded our understanding of AD from the perspective of neuroimaging and neuropathology (Ossenkoppele et al., 2022), with the pathological hallmarks of amyloid *β* (Aβ) protein accumulation outside neurons and twisted strands of the tau tangles inside neurons being identified (Gibbons et al., 2019; Cho et al., 2022). Nevertheless, the diagnostic modalities and novel therapeutic agents targeting AD remain unsatisfactory due to the high clinical heterogeneity and complex genomic classification. Moreover, previous studies investigating biomarkers associated with AD have been limited owing to the small sample sizes and inadequate bioinformatic tools. This underscores the need to unravel the molecular classification of AD and identify novel therapeutic targets. By doing so, we can establish a framework for making personalized clinical decisions in the management of AD.

The last decade has witnessed unprecedented strides in terms of the characterization of programmed cell deaths as evidenced by the identification of monitoring biomarkers and molecular targets for diseases (Wang et al., 2023). Among them, necroptosis is one of the types of programmed cell death which shares the features of apoptosis and necrosis (Dhuriya and Sharma, 2018), and it was first identified in the study of AD by Caccamo et al. (2017). Specifically, the Receptor-interacting serine/threonine-protein kinase 1 (RIPK1) and activation of receptor-interacting protein kinase 3 (RIPK3) are the core taches of necroptosis, which can phosphorylate and activate the mixed lineage kinase domain-like pseudokinase (MLKL), thereby contribute to plasma-membrane permeabilization and cell death (Tanzer et al., 2021). Research has revealed the mechanisms by which necroptosis affects multiple neurological and neurodegenerative disorders, including amyotrophic lateral sclerosis (Wang et al., 2020), Parkinson’s disease (Oñate et al., 2020) and Huntington’s disease (Zhu et al., 2011). In addition, there is compelling evidence supporting the involvement of necroptosis in the pathogenesis of AD (Koper et al., 2020; Lee et al., 2021), with substantial *in vivo* data demonstrating its participation in development of cognitive deficits in APP/PS1 mice (Caccamo et al., 2017). Clinical studies have shown that necroptosis is activated in AD patients (Jayaraman et al., 2021). The feasibility of necroptosis-related genes (NRGs) as the diagnostic and therapeutic modalities for AD have been documented (Ofengeim et al., 2017). Hence, deciphering the molecular classification and genomic heterogeneity of AD populations based on necroptosis and its associated gene drivers is of great significance to improving our understanding of the pathogenesis and development of AD.

**Figure 1:**
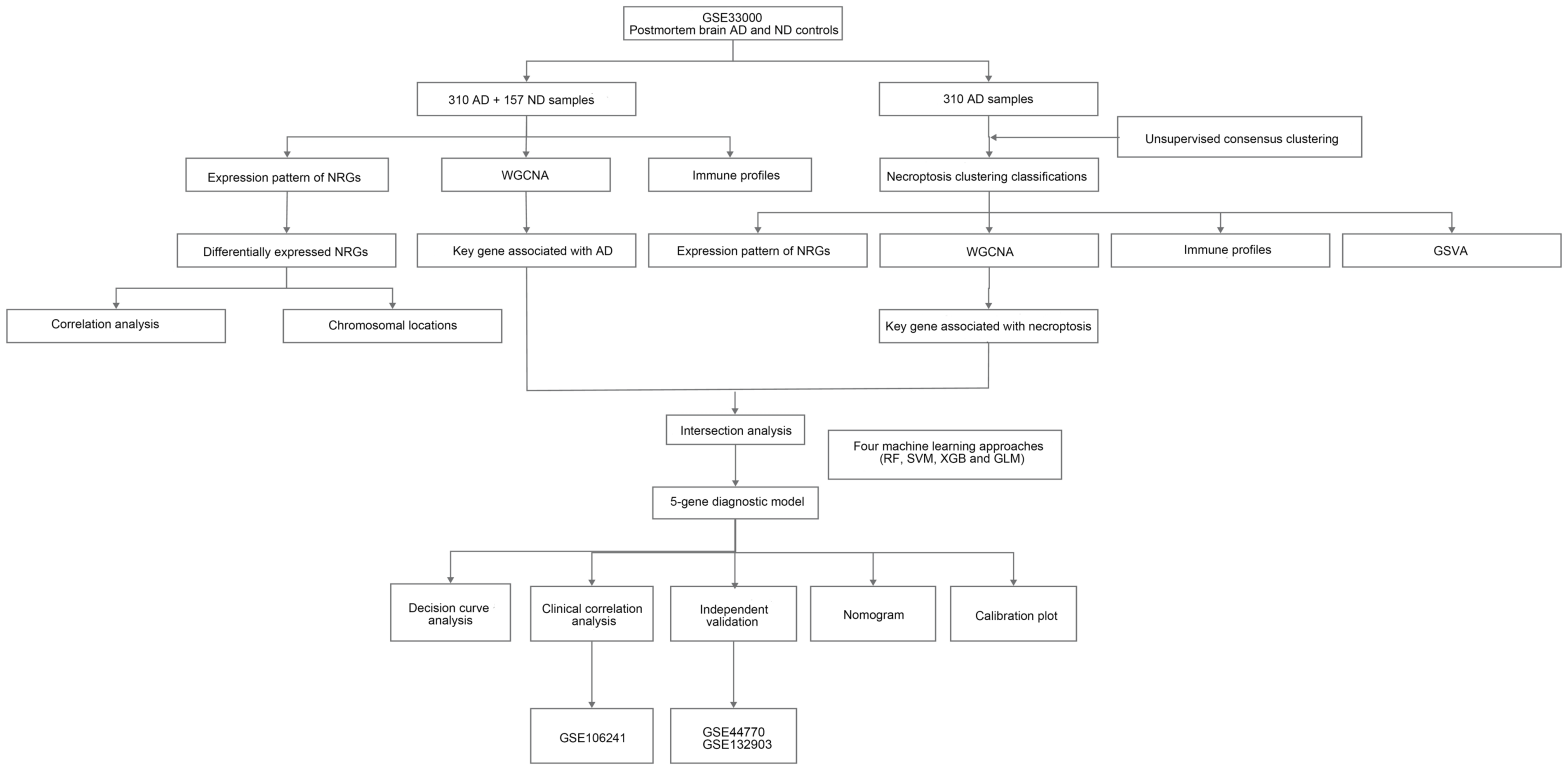
The flow chart of the study. We first investigated necroptosis-related gene (NRG) expression profiles and identified differential expression between Alzheimer’s disease (AD) and non-demented (ND) controls. Then, we explored immune profiles and conducted consensus clustering on AD samples, followed by identifications of key genes related to necroptosis clusters and AD using weighted gene co-expression network analysis (WGCNA) algorithm. Next, we developed a diagnostic model for AD using diverse machine learning approaches and assessed its performance.

In this study, we investigated the expression profiles of NRGs, identified differentially expressed NRGs between AD and non-demented (ND) controls, and then explored the immune profiles of the samples. Next, AD samples in the training set were extracted and subjected to consensus clustering based on differentially expressed NRGs. Results revealed that the patients could be classified into two necroptosis clusters, each with distinct immune landscape and enriched pathways. Subsequently, key genes related to necroptosis clusters were identified using WGCNA algorithm, and intersected with the key gene related to AD, thereby obtaining the shared genes between module-related genes in AD and in necroptosis clusters. Subsequently, a diagnostic model for AD was developed by comparing various types of machine learning approaches, with the nomogram, and its discrimination performance and stabilities in the diagnosis of AD were determined using the calibration plot, decision curve analysis (DCA), and independent validation datasets. Furthermore, we performed correlation analyses for the hub genes included in the diagnostic model with the age and major risk factors influencing the generation of Aβ, and further elucidated its correlation with the pathologic hallmarks of AD. The flow chart of the study was shown in Figure 1.

## Materials and methods

### Data acquisition and sample information

Microarray data of transcription profiles associated with AD were acquired from the National Center for Biotechnology Information (NCBI) Gene Expression Omnibus (GEO) database (accessed on December 31, 2022).^1^ GSE33000, containing RNA-seq data of prefrontal cortex from 310 postmortem brain of patients with AD and 157 ND samples, was used as the training set, and used to construct a diagnostic model for AD. Given that our analysis focused solely on AD, we excluded samples related to Huntington’s disease from this dataset. GSE44770 and GSE132903 datasets were used as the independent validation datasets to evaluate the predictive performance of the constructed model, both of which contain transcription profiles of postmortem brain of AD and healthy controls. In addition, the GSE106241 dataset was retrieved from the GEO database to investigate the association between the hub genes in the diagnostic model and clinical characteristics of AD patients. All cases were retained for our analysis in such three datasets. The detailed information of datasets used are presented in Table 1. Of note, we conducted a power calculation to determine the minimum required sample size for a two-sample t-test comparing AD and ND samples. We assumed a significance level of 0.05 and a desired power of 0.8. The effect size (Cohen’s d) was set to 0.5. Based on these parameters, the power calculations indicated that a minimum sample size of 64 samples per group is needed to achieve the specified power and significance level, which suggested that the sample sizes in the training and validation datasets were adequate.

**Table 1:** Detailed information for the datasets used in this study.

Accession number	Platform	Sample size	Sample source	Female: Male	Mean age of AD (year)	Mean age of NC (year)
GSE33000	GPL4372	310 AD and 157 NC	Prefrontal cortex	209:258	80.6	63.52
GSE44770	GPL4372	129 AD and 101 NC	Dorsolateral prefrontal cortex, visual cortex and cerebellum	86:144	80.15	62.12
GSE132903	GPL10558	97 AD and 98 NC	Middle temporal gyrus	96:99	85.02	84.98
GSE106241	GPL24170	60 AD	Inferior temporal cortex	42:18	80.67	/

AD, Alzheimer’s disease; ND, non-demented controls.

### Identification and analysis of differentially expressed necroptosis-related genes

Necroptosis-related genes were retrieved from the Deathbase database (Díez et al., 2010; accessed on December 31, 2022),^2^ the Gene Set Enrichment Analysis (Subramanian et al., 2005; GSEA, accessed on December 31, 2022),^3^ and GeneCards database (Stelzer et al., 2016) with a relevance score threshold of 1.5 (accessed on December 31, 2022).^4^ The search was conducted using the term “necroptosis,” which yielded 46 NRGs. Next, the R package “limma” was used to identify differentially expressed NRGs between AD and ND samples, and results of differentially expressed NRGs were visualized using R packages “ggpubr” and “pheatmap,” while the correlation between varying NRGs was visualized using “circlize” packages.

### Classification of Alzheimer’s disease samples using consensus clustering

Based on the differentially expressed NRGs, we employed the “ConsensusClusterPlus” package (Wilkerson and Hayes, 2010) to cluster the AD samples in the training set. This allowed us to determine the optimal number of clusters through the analysis of consensus matrix plots, consensus cumulative distribution function (CDF) plots, and trace plots. Subsequently, principal component analysis (PCA) was conducted to visualize the distribution of samples based on necroptosis-related patterns, focusing on the first two principal components after clustering.

### Gene Set variation analysis (GSVA)

Gene set variation analysis enrichment analysis was performed to determine the biological processes and pathways related to the disparate clusters using “GSVA” packages, based on the gene sets of “c2.cp.kegg.v7.2.symbols” provided by the Molecular Signatures Database (MsigDB).^5^ The most significant terms with *p*-value less than 0.05 calculated by student’s t-test were displayed on a barplot, with red and blue representing up- and downregulated pathways, respectively.

### Identification of key genes using weighted gene co-expression network analysis (WGCNA)

To explore the connections between gene modules and disease traits in the context of AD, we employed WGCNA in conjunction with hierarchical clustering. This analysis, performed using the “WGCNA” package, enabled us to identify crucial genes that are closely associated with the pathogenesis of AD. Briefly, the genes with the top 25% variance were extracted from the GSE33000, and used to cluster the samples and remove outliers. Next, Pearson’s correlation value between varying gene pairs was calculated, and utilized to construct a similarity matrix, the latter of which was converted into the adjacent matrix with a suitable soft threshold power. Furthermore, a topological overlap matrix (TOM) was generated, and genes that shared similar expression pattern were categorized into disparate modules via a dynamic tree-cutting algorithm using minimum module size of 100 genes a threshold. Finally, we defined the hub genes within each module as those exhibiting a gene significance (GS) value greater than 0.2 and a module membership (MM) value exceeding 0.6.

### Analysis of immune cell infiltration

CIBERSORT algorithm was used to identify the relative composition of 22 immune cells based on expression profiles of the samples in GSE33000 dataset, which is a widely used analytical tool that is based on linear support vector regression principle for deconvolution analysis (Chen et al., 2021, 2022). The relative compositions of 22 immune cells in different groups and their correlations with NRGs were performed and visualized using “ggplot2” and “ggpubr” packages.

### Establishment and verification of diagnostic model for Alzheimer’s disease using various machine learning algorithms

After intersection analysis of the genes in the most significant modules in WGCNA, “caret” R package was utilized to divide the samples into two datasets using the createDataPartition function, and a 70 to 30% split was applied, ensuring a representative distribution of samples across the sets. Then, the key genes with high diagnostic potential for AD were identified using four machine learning algorithms, including Random Forest (RF), Support Vector Machines (SVM), Extreme Gradient Boosting (XGB), and Generalized Linear Model (GLM), were applied to rank features by importance in the training set via “kernlab,” “randomForest,” “xgboost” R packages. In the RF algorithm, we implemented the train function with the “rf” method, and trainControl function was employed to establish the settings for repeated cross-validation. Additionally, we defined the p_fun function to predict class probabilities. For the SVM model, we employed the train function with the “svmRadial” method for training, and the parameter prob.model was set to TRUE to enable probability modeling. In the case of the XGB model, we constructed it using the train function with the “xgbDART” method, and the cross-validation settings were specified via the trainControl function. Similarly, we used the train function with the “glm” method to create the GLM model. To handle binary classification, the family parameter was set to “binomial.” Besides, diseases phenotype was used as the response variable, whereas the genes obtained by WGCNA were used as the explanatory variable. Next, the explain function of “DALEX” R package was employed to perform exploratory analysis for the model and plot function was used to generate the cumulative residual distribution map and residual boxplot, thereby identifying the optimal diagnostic model combined with time-dependent receiver operating characteristic (ROC) curve with evaluation of the area under the ROC curve (AUC) through “pROC” package. Finally, five most important variables were used to construct a five-gene diagnostic model, and such a diagnostic model was validated through multiple validation sets.

### Statistical analysis

The nonparametric Wilcoxon test was used to compare two groups of non-normally distributed data, while student’s *t*-test was applied to analyze normally distributed data. Correlation analyses were conducted using the Spearman correlation test. These analyses were performed using the R software 4.1.2, and *p* < 0.05 was considered statistically significant.

## Results

### Aberrant expression pattern of necroptosis-related genes in Alzheimer’s disease

**Figure 2:**
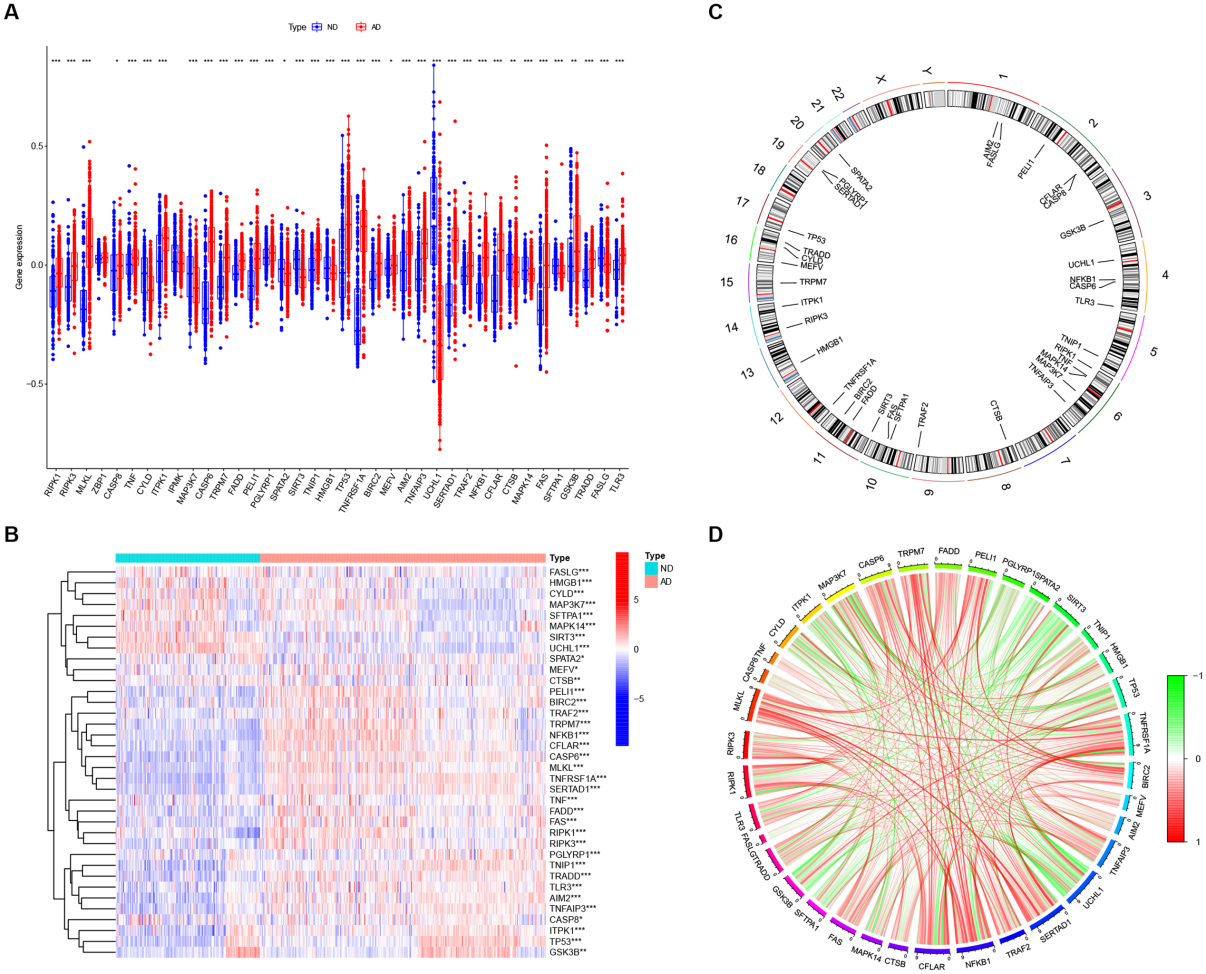
The expression patterns of NRGs in AD. **(A)** Boxplots displaying the expression patterns of 36 differentially expressed NRGs between AD and ND samples. *p < 0.05, **p < 0.01, and ***p < 0.001. **(B)** Heatmap showing the relative expression levels of 36 detected and differentially expressed NRGs. *p < 0.05, **p < 0.01, and ***p < 0.001. **(C)** Chromosomal locations of 36 differentially expressed NRGs. **(D)** Correlation analysis for the 36 differentially expressed NRGs, with red and green lines indicating positive and negative correlations, respectively.

We searched for previously reported NRGs from public databases and identified 46 NRGs. To investigate the expression profiles of NRGs in AD, 310 postmortem brain samples of patients with AD and 157 ND samples in the GSE33000 were analyzed after log2 transformation. Results showed that 36 out of the 46 NRGs were differentially expressed in AD samples. The expression levels of RIPK1, RIPK3, MLKL, CASP8, TNF, CASP6, TRPM7, FADD, PELI1, PGLYRP1, TNIP1, TP53, TNFRSF1A, BIRC2, MEFV, AIM2, TNFAIP3, SERTAD1, TRAF2, NFKB1, CFLAR, FAS, GSK3B, TRADD, and TLR3 were significantly enhanced in AD samples, whereas that of CYLD, ITPK1, MAP3K7, SPATA2, SIRT3, HMGB1, UCHL1, CTSB, MAPK14, SFTPA1, and FASLG were downregulated in AD samples (Figure 2A), and a heatmap displaying the disparate expression levels of such differentially expressed NRGs between postmortem brain of patients with AD and ND was constructed (Figure 2B). The chromosomal locations of the differentially expressed NRGs are shown in Figure 2C. Moreover, the correlations among these differentially expressed NRGs were diverse (Figure 2D), further revealing their potential interactive regulation of AD.

**Figure 3:**
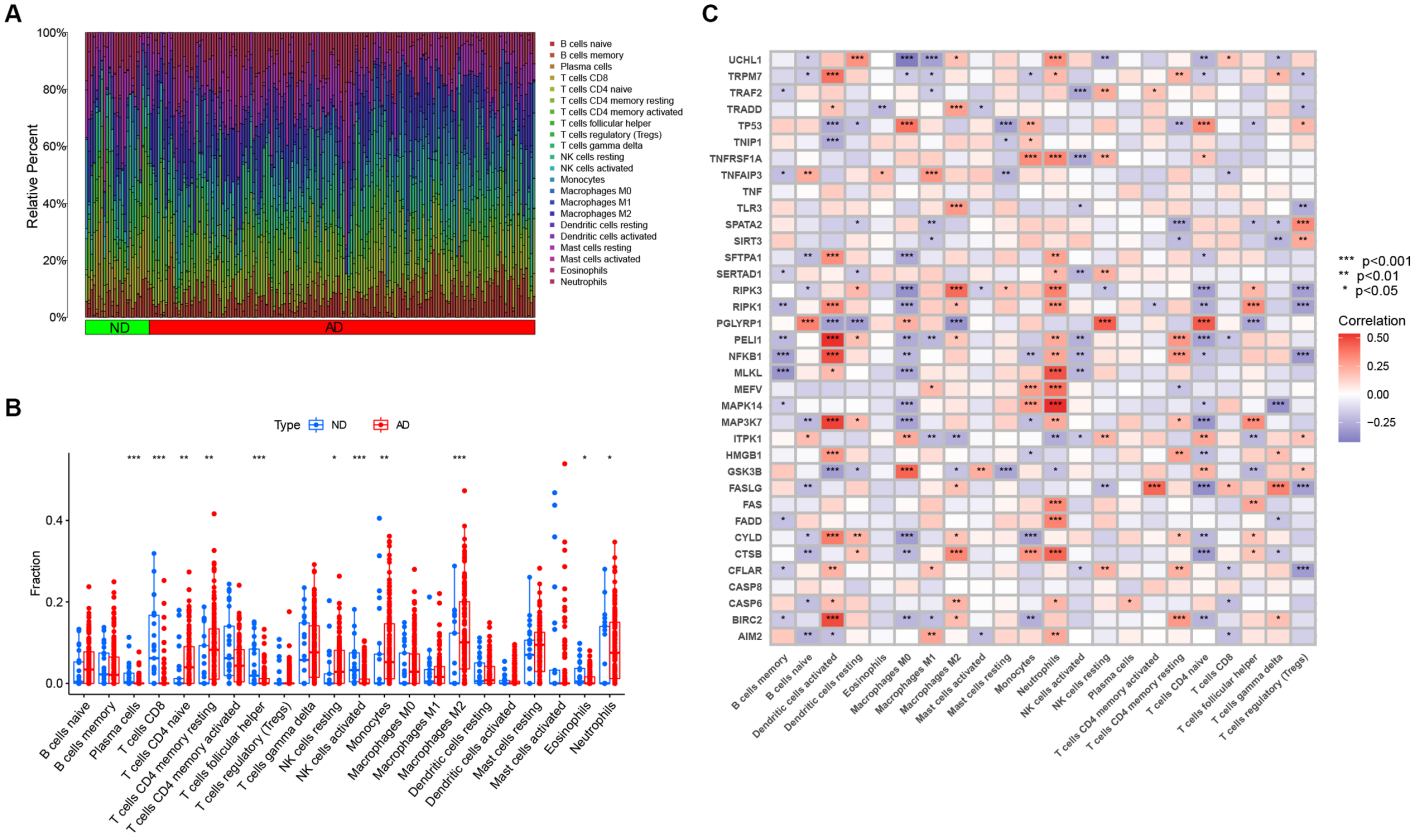
The immune landscape of AD and ND samples evaluated using the CIBERSORT algorithm. **(A)** Heatmap showing the relative proportions of infiltrating immune cells in AD and ND samples from the GSE33000 dataset. **(B)** Barplot showing the differential analysis of various infiltrating immune cells between AD and ND samples. *p < 0.05, **p < 0.01, and ***p < 0.001. **(C)** Correlation analysis between 36 differentially expressed NRGs and infiltrating immune cells. *p < 0.05, **p < 0.01, and ***p < 0.001.

Studies have demonstrated that the innate and adaptive immune responses participate in the progression of AD (Lee et al., 2021), and various cellular identities in the immune microenvironments are associated with the neuropathological hallmarks of AD (e.g., Aβ protein deposits, and neurofibrillary tangles; Baruch et al., 2015; Zenaro et al., 2015; Marsh et al., 2016). Therefore, immune cells are promising therapeutic targets for the control of AD. Consequently, we employed the CIBERSOTR algorithm to assess the relative proportions of immune cells of samples in the GSE33000, and visualized the results using a heatmap (Figure 3A). The relative proportions of multiple infiltrating immune cell varied among the groups (Figure 3B). Alzheimer’s disease samples exhibited higher proportions of naive CD4 T cells, resting memory CD4 T cells, resting NK cells, monocytes, neutrophils and macrophages M2, whereas ND samples had higher levels of plasma cells, CD8^+^ T cells, follicular helper T cells, activated NK cells, and eosinophils. Subsequent correlation analysis revealed that the expression levels of the afore-mentioned differentially expressed NRGs were closely linked with the abundance of immune cells in the local environment (Figure 3C), especially with neutrophils and macrophages M0, which are strongly associated with 20 and 18 immune cell subtypes, respectively. This further confirmed the strong cooccurrence between NRGs and the immune cell subpopulations in the local environment of brain, which create bridges between necroptosis and AD occurrence and development (Zenaro et al., 2015; Salvadores et al., 2022). In conclusion, these results demonstrated that NRGs contribute to the development of AD and have profound influence in the immune microenvironment.

### Construction of unsupervised necroptosis clusters in Alzheimer’s disease samples and investigation of the mechanisms

**Figure 4:**
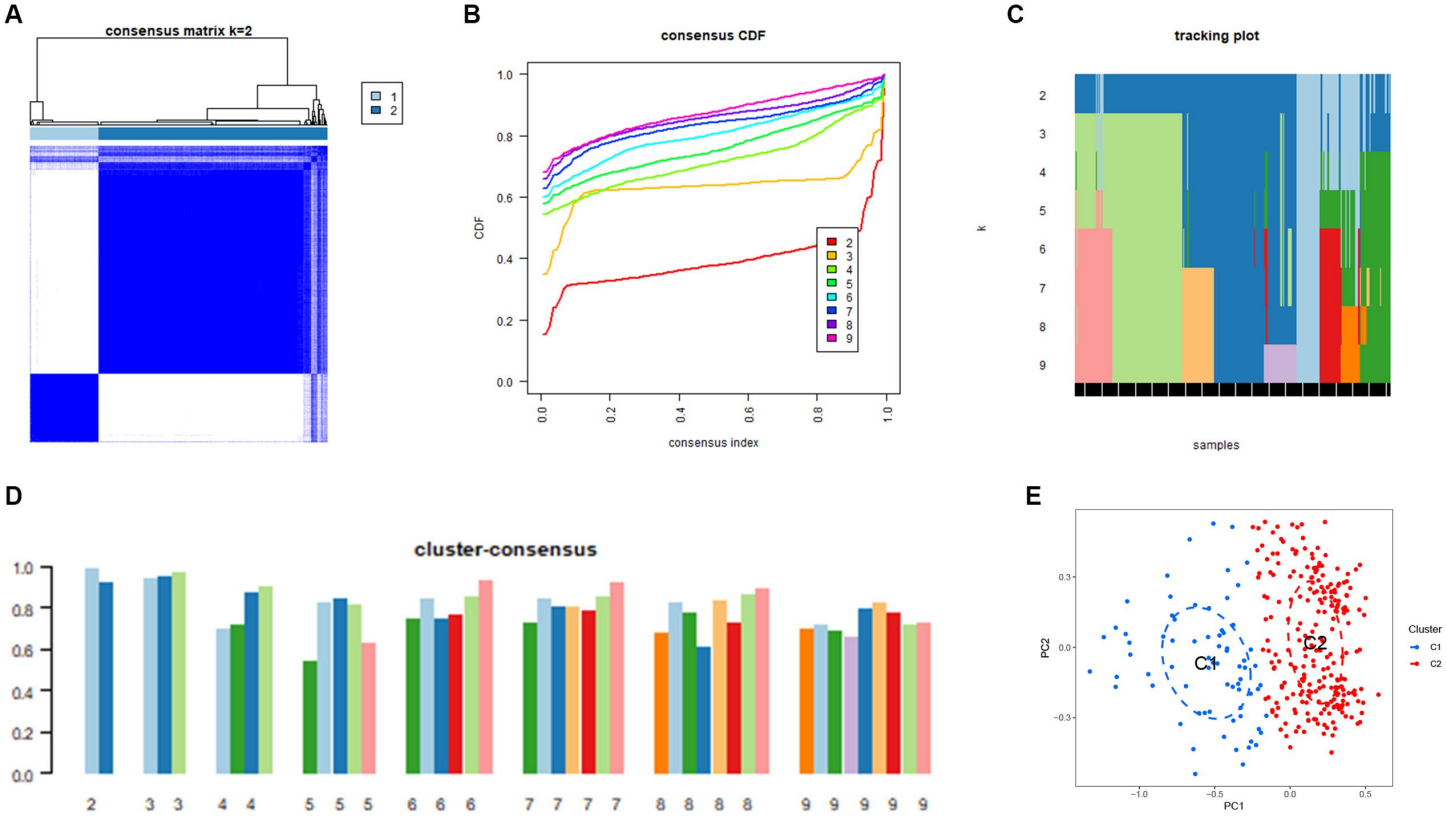
Construction of unsupervised necroptosis clusters of AD samples. **(A)** Unsupervised consensus clustering matrix when *k* = 2. **(B)** Consensus CDF curves showing minimal fluctuations at different consensus indexes. **(C)** Trace plot illustrating the clustering of each sample when *k* values were set from 2 to 9. **(D)** The scores of consensus clustering when *k* values were set from 2 to 9. **(E)** Principal component analysis (PCA) analysis showing the distribution of two necroptosis clusters identified using unsupervised consensus clustering.

**Figure 5:**
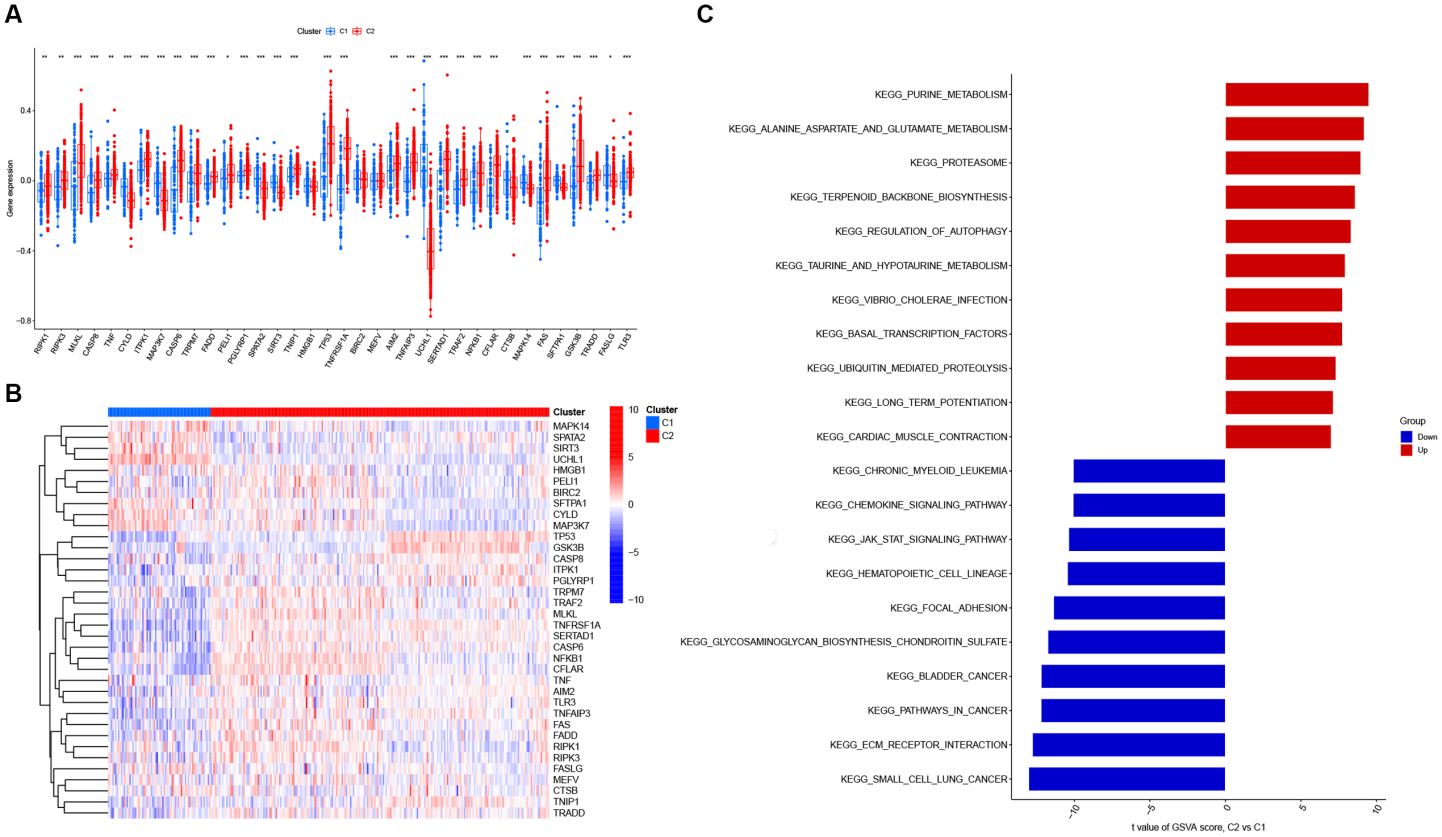
The expression patterns of NRGs in two necroptosis clusters identified by unsupervised consensus clustering. **(A)** Boxplots displaying that 32 out of 36 NRGs were differentially expressed between two necroptosis clusters. *p < 0.05, **p < 0.01, and ***p < 0.001. **(B)** Heatmap presenting the relative expression levels of 36 NRGs in two necroptosis clusters of C1 and C2. **(C)** Differences in hallmark pathway activities between two necroptosis clusters of C1 and C2 samples ranked by t-value of gene set variation analysis (GSVA) enrichment analysis.

Next, 310 AD samples in the training set were extracted and subjected to consensus clustering based on the differentially expressed NRGs. It was observed that the most optimal number of clusters was two in consensus matrix plots (*k* = 2; Figure 4A), with the consensus CDF curves showing minimal fluctuations at different consensus indexes (Figure 4B) and the trace plot (Figure 4C) representing its stability. Moreover, when the value of k was set to 2 (Figure 4D), we observed a high consistency score exceeding 0.9 for each cluster. Based on this finding, we divided the 310 AD samples into two distinct clusters: Cluster 1 (C1, *n* = 72) and Cluster 2 (C2, *n* = 238), with the clinical characterization of necroptosis clusters of AD samples being displayed in Table 2. Subsequently, a PCA revealed a clear distinction between these two clusters (Figure 4E). To further investigate the molecular underpinnings between disparate necroptosis clusters, we then performed systematic analyses of the necroptosis clusters constructed previously. Results showed that multiple NRGs were differentially expressed between C1 and C2. Specifically, 32 out of the 36 NRGs were differentially expressed between two necroptosis clusters (Figure 5A), and a heatmap to display the relative expression patterns of such 36 NRGs in the AD samples was constructed (Figure 5B). Furthermore, GSVA analysis was performed to investigate the pathways involved between clusters. It was noted that metabolism-related pathways, spanning purine metabolism, alanine aspartate and glutamate metabolism, as well as taurine and hypotaurine metabolism were upregulated in C2. Conversely, immune-related pathways, including chemokine signaling pathway and JAK STAT signaling pathway, were enriched in the C1 (Figure 5C). Likewise, the CIBERSORT algorithm was employed to estimate the infiltrating immune cells in the two clusters, and a barplot showing the relative abundance of infiltrating immune cell in samples was established (Supplementary Figure 1A). A boxplot (Supplementary Figure 1B) showing the comparisons of various infiltrating immune cells was plotted. Surprisingly, six types of infiltrating immune cells exhibited significant differences in relative abundance, most of which were enriched in C2, including the naive B cells, naive cells CD4, resting NK cells, Macrophages M0 and M1. In contrast, activated NK cells were more abundant in C1. These findings demonstrate differences between the two necroptosis clusters, and reveal the mechanism underlying their association.

**Table 2:** Clinical characterization of necroptosis clusters of AD samples.

Cluster number	Sample size	Female: Male	Mean age of AD (year)
Cluster 1	72	28:44	80.60
Cluster 2	238	147:91	80.71

AD, Alzheimer’s disease.

### Construction of a co-expression network and identifications of key gene associated with Alzheimer’s disease and necroptosis using weighted gene co-expression network analysis

**Figure 6:**
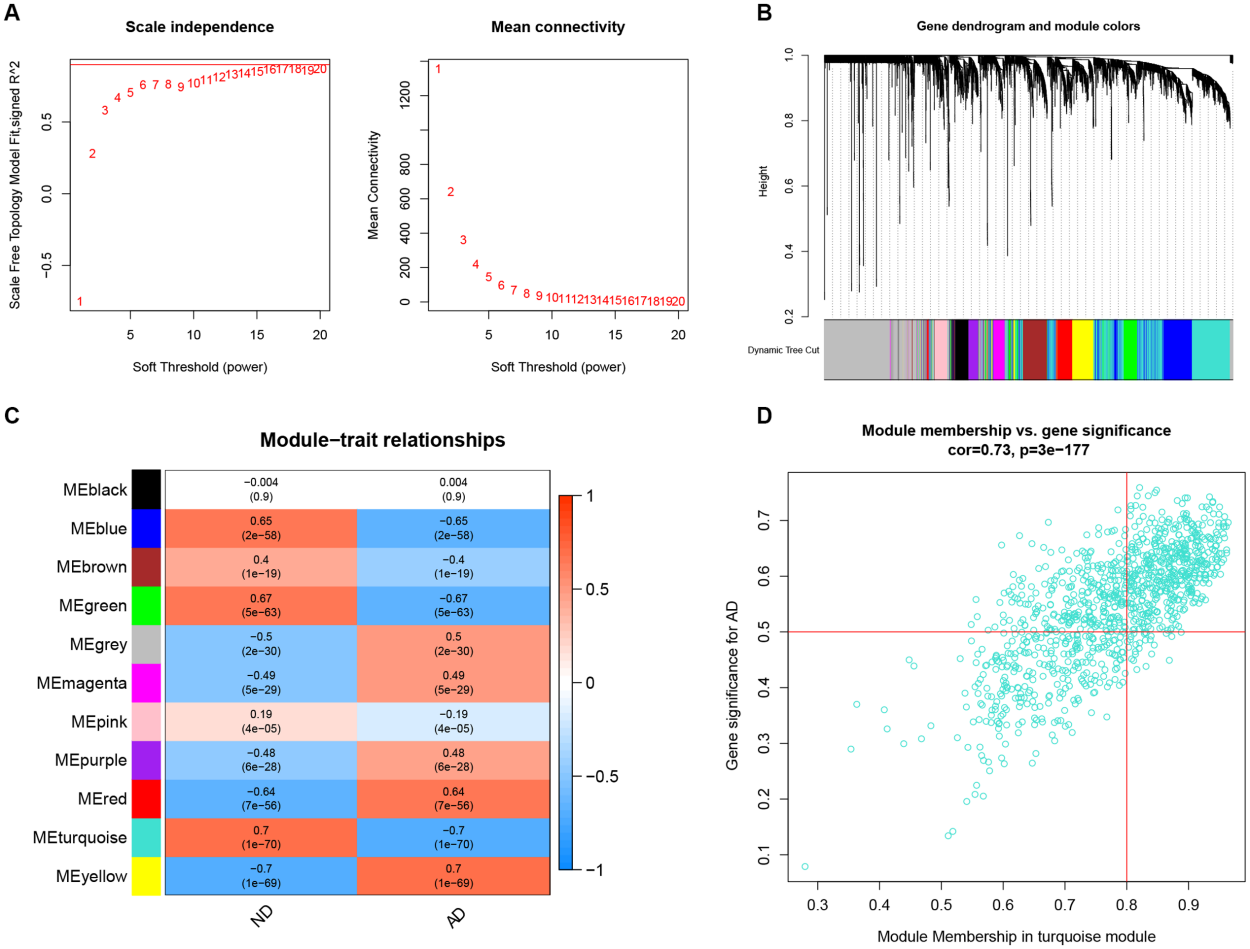
Construction of co-expression network and identifications of key gene associated with AD using WGCNA. **(A)** Selection of the best soft threshold power *β*. **(B)** Clustering dendrogram of the co-expression network, with various colors representing disparate co-expression modules. **(C)** Correlation analysis between disparate co-expression modules and clinical traits. **(D)** Correlation of module membership (MM) and gene significance (GS) in the turquoise module with the AD.

To identify the key genes linked with AD, the WGCNA algorithm was utilized to construct a co-expression network and modules for AD and ND subjects as previously described. After extraction of the genes with the top 25% variance and removal of the abnormal samples in the GSE33000 dataset, a scale-free network was established with a soft threshold of 15 and the scale-free R^2^ was equal to 0.85 (Figure 6A). In total, 11 disparate co-expression modules were identified (Figure 6B). Among them, the turquoise module showed the highest correlation and most significant *p* value with AD (*r* = 0.7, *p* = 1e−70; Figure 6C), and the 983 genes in such module were obtained after the analysis, with the scatter plot showing a significantly positive correlations between the turquoise module and corresponding genes (Figure 6D).

**Figure 7:**
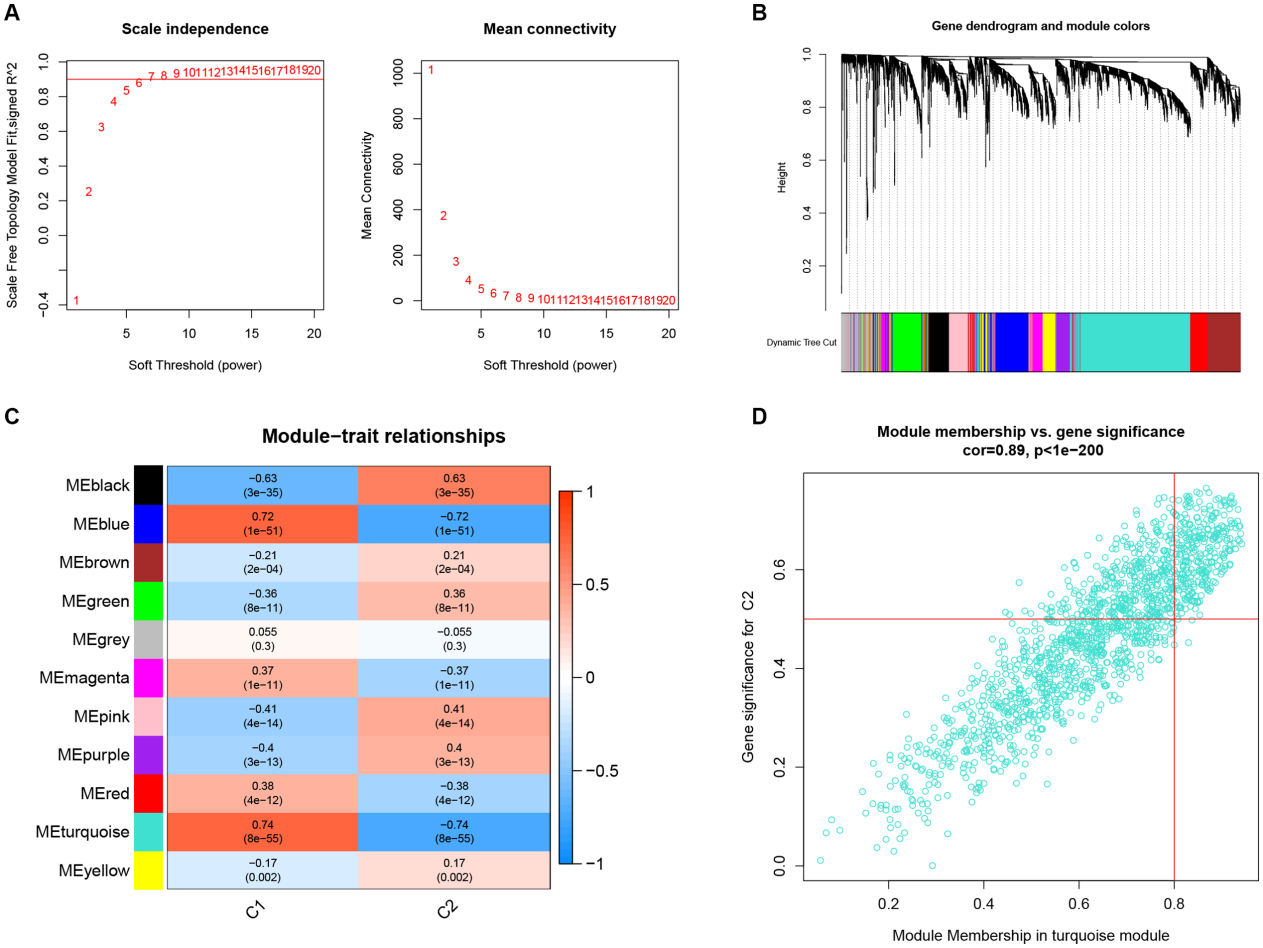
Construction of co-expression network and identification of key gene associated with necroptosis using WGCNA. **(A)** Selection of the best soft threshold power *β*. **(B)** Clustering dendrogram of the co-expression network, with various colors representing disparate co-expression modules. **(C)** Correlation analysis between disparate co-expression modules and clinical traits. **(D)** Correlation of MM and GS in the turquoise module with C2.

In further analyses, we employed the WGCNA algorithm to identify the key gene related to necroptosis clusters with the AD objectives in GSE33000. *β* = 6 and *R*^2^ = 0.87 were used as the most suitable soft threshold to establish a scale-free network (Figures 7A,B). Turquoise module showed the highest correlation and most significant *p* value with necroptosis clusters (*r* = 0.74, *p* = 8e−55; Figure 7C), and the 877 genes in turquoise module were selected for the subsequent analysis, especially for the genes that showed significant correlation with such module (Figure 7D).

**Figure 8:**
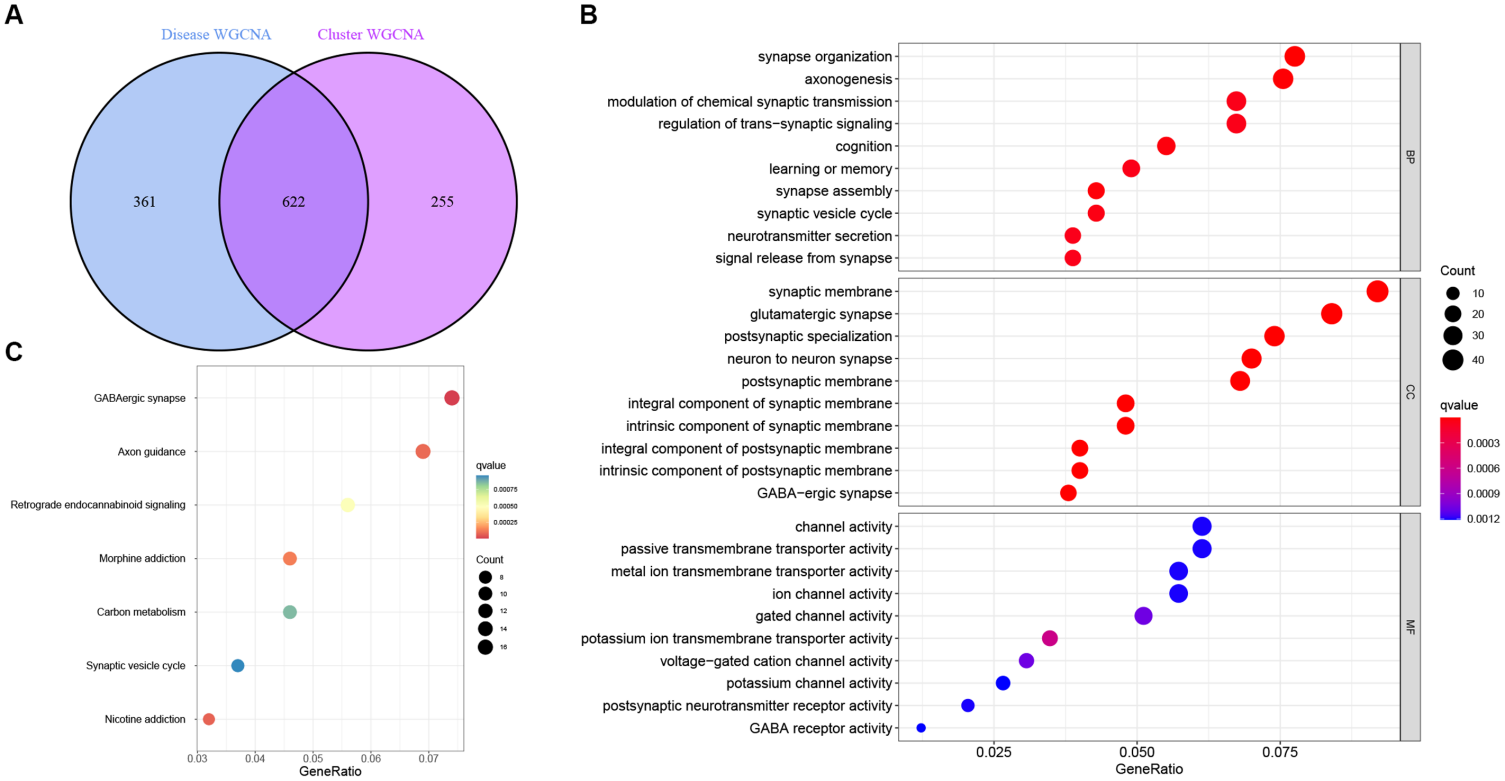
Identification and functional enrichment analysis of the key genes identified by WGCNA. **(A)** Analysis of the intersection between module-related genes associated with necroptosis clusters and module-related genes associated with AD. **(B)** Gene ontology (GO) functional enrichment analysis of the shared genes obtained in the intersection analysis. **(C)** Kyoto Encyclopedia of Genes and Genomes (KEGG) pathway signaling enrichment analysis of the shared genes obtained in the intersection analysis.

Next, intersectional analysis of the key genes obtained in WGCNA was conducted, yielding 622 shared genes between module-related genes in AD and ND as well as module-related genes in necroptosis clusters (Figure 8A). Specific gene lists are provided in Supplementary Table S1. Subsequent Gene ontology (GO) functional enrichment analysis indicated that such shared genes were mainly enriched in the synaptic development, neurotransmitter transport, neurogenesis, and axonogenesis, suggesting their key roles in the preservation of cognitive and neurologic function and homeostasis (Figure 8B). Similarly, the Kyoto Encyclopedia of Genes and Genomes (KEGG) pathway signaling enrichment analysis demonstrated that such shared genes were mainly enriched in the GABAergic synapse, axon guidance, and synaptic vesicle cycle pathways (Figure 8C).

### Construction of a diagnostic model for Alzheimer’s disease using various types of machine learning approaches

**Figure 9:**
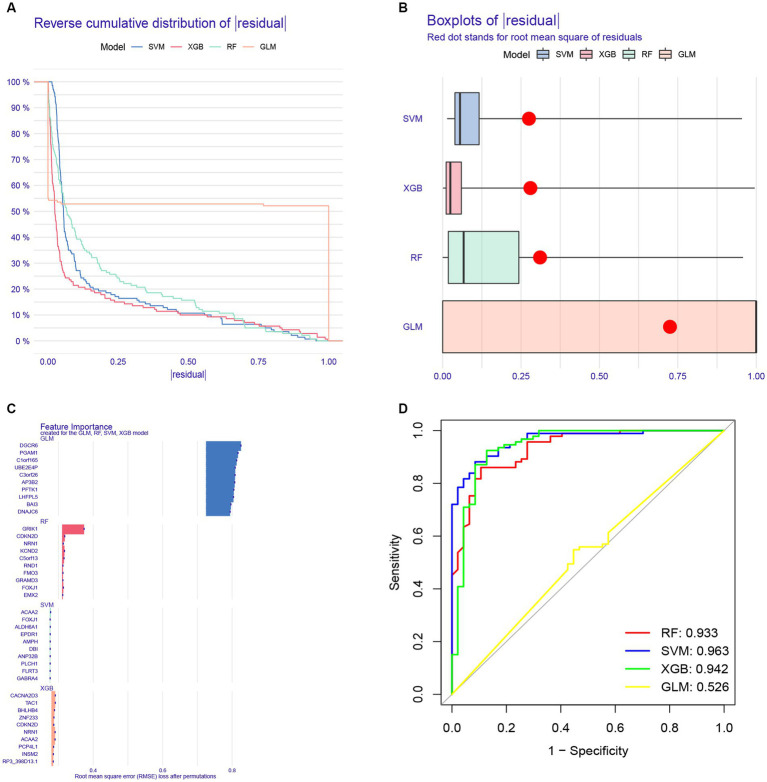
Construction of a diagnostic model for AD using different machine learning methods. **(A)** Cumulative residual distribution map of four machine learning approaches. **(B)** Boxplots presenting the residuals of each machine learning approaches, with red dot indicating the root mean square error (RMSE). **(C)** Top 10 variables ranked by RMSE in four machine learning approaches. **(D)** Receiver operating characteristic (ROC) curve of four machine learning approaches based on 30% of the samples in the GSE33000 dataset.

To further analyze the 622 shared genes obtained in WGCNA and identify the hub genes with diagnostic potential for AD, four machine learning approaches, including RF, SVM, XGB and GLM, were used to construct the diagnostic models using 70% of the samples in GSE33000. As shown in the cumulative residual distribution map (Figure 9A) and residual boxplot (Figure 9B) of the four machine algorithms, XGB and SVM exhibited a comparatively smaller size in residual values, demonstrating the reliability of the constructed models. Figure 9C shows the top 10 variables for each model ranked by root mean square error (RMSE). Next, ROC curve was plotted to evaluate the diagnostic performance of each model using the remaining 30% of the samples in GSE33000 (Figure 9D). Notably, three machine learning models demonstrated excellent discrimination performance, as evidenced with an AUC > 0.9. Based upon the predictive ability and reliability of the models constructed by four machine learning approaches, XGB was considered as the most optimal diagnostic model for the AD, and the five most important variables including ACAA2, BHLHB4, CACNA2D3, NRN1, and TAC1 were used to construct a five-gene diagnostic model for AD.

**Figure 10:**
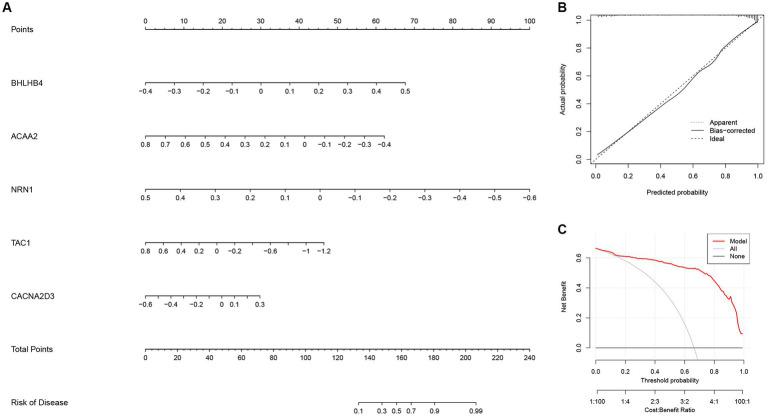
The diagnostic model for AD constructed with the five most important variables in the Extreme Gradient Boosting (XGB) model. **(A)** Construction of the nomogram for predicting the risk of AD based on the five most important variables in the XGB model. **(B)** The calibration plot showing the predictive accuracy of the actual probability and predicted probability. **(C)** Decision curve analysis (DCA) showing the predictive efficiency of the constructed model.

Next, a nomogram based on ACAA2, BHLHB4, CACNA2D3, NRN1, and TAC1 was established (Figure 10A), and calibration plot showed a good predictive accuracy between the actual probability and predicted probability (Figure 10B). Furthermore, DCA confirmed that the model had high significance in clinical decision-making (Figure 10C). In summary, we used machine algorithm to establish a diagnostic model for AD based on the shared genes between module-related genes in disease and module-related genes in necroptosis-related clusters of WGCNA. The performance of the model in diagnosing AD was satisfactory.

### Validation of the five-gene diagnostic model in external cohorts and its clinical correlation analysis

**Figure 11:**
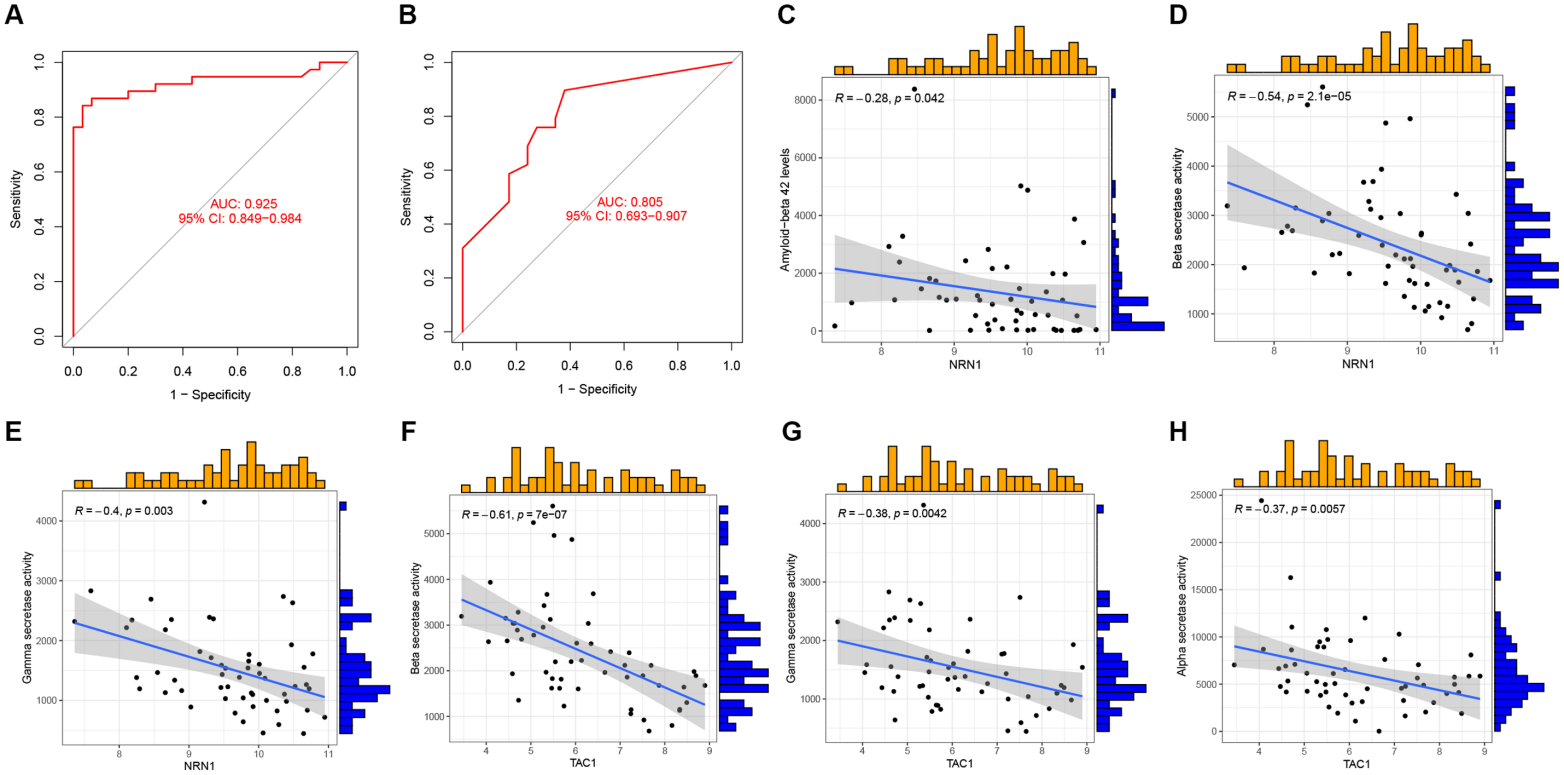
Validation of the five-gene diagnostic model in external cohorts and its correlation with clinical features. **(A)** ROC analyses evaluating the predictive potential of the five-gene diagnostic model using GSE44770 as the independent validation dataset. **(B)** ROC analyses exploring the predictive potential of the five-gene diagnostic model using GSE132903 as the external validation dataset. **(C–E)** Association of NRN1 with Aβ 42 levels, *β*-, and *γ*-secretase activities. **(F–H)** Association of TAC1 with *β*-, *γ*-, α-secretase activities.

Next, GSE44770 and GSE132903 datasets were used as the independent validation datasets to determine the predictive performance of the five-gene diagnostic model. The ROC analyses were conducted which yielded the AUC value of 0.925 in the GSE44770 (Figure 11A), and the AUC value of 0.805 in the GSE132903 (Figure 11B). These results indicated that the constructed model achieved excellent discrimination performance and stability, demonstrating a high clinical significance. Next, we performed correlation analyses of the expression levels of the hub genes included in the diagnostic model with the age and key factors affecting the generation of Aβ, including Aβ 42 (Marttinen et al., 2019), α-, β-, and γ-secretase activity in GSE106241 dataset (Hur, 2022). Intriguingly, we discovered that NRN1 was negatively correlated with Aβ42, β-, and γ-secretase activities, suggesting it may be a potential indicator for determining the severity of pathologic alterations in AD (Figures 11C–E). Similar observations were made for TAC1 (Figures 11F–H). Similarly, CACNA2D3 was exclusively associated with β-secretase activity (Supplementary Figure 2A), whereas the expression levels of ACAA2 and NRN1 tended to be higher in AD cases (Supplementary Figures 2B–C). In addition, we validated the diagnostic model of AD in other independent cohorts, and confirmed its outstanding diagnostic performance in AD and close relationship with the pathologic hallmarks of AD.

## Discussion

Since the discovery of AD by Dr. Alois Alzheimer of Germany in the early 20th century (Bondi et al., 2017), the ensuing century has witnessed unprecedented strides with regard to our understanding of AD at the pathological, clinical and biological levels (Scheltens et al., 2021). Despite the typical histopathological and neuroimaging alterations, substantial heterogeneity has been demonstrated in the age of onset, clinical manifestations and pathological changes of AD (Duara and Barker, 2022). Furthermore, the presence of diverse phenotypes of AD can lead to its misdiagnosis. This is primarily due to the absence of typical clinical symptoms and pathological features, further complicating accurate identification and classification (Graff-Radford et al., 2021). This also explains the failure of majority of drug candidates during development and clinical trials (van Bokhoven et al., 2021). Although substantial progress has been made over the past decades, a review of available publications suggests that there is need to further dissect the heterogeneity and classification of disease subtypes of AD (Iturria-Medina et al., 2022).

To close the gap existing in our understanding of the molecular mechanisms of AD, we systematically and comprehensively investigated the transcriptomic profiles of necroptosis regulators between AD and ND samples, to identify the aberrant expression patterns of NRGs in AD. Our findings demonstrate the underlying connections of necroptosis in the pathogenesis of AD. Intriguingly, we uncovered the specific immune cell landscape in the microenvironment of AD, and the subtypes of T cells were significantly varied between AD and ND individuals, implying that T cells play key roles in the progression of AD. Specifically, AD individuals exhibited high relative abundance of naive and resting memory CD4 T cells, whereas ND samples had markedly higher number of CD8^+^ and follicular helper T cells. Such alterations were also reported in animal models (Chen et al., 2023) and cerebrospinal fluid of AD patients (Busse et al., 2021), in which circulating T cells in AD patients exhibited a more activated phenotype than those of healthy individuals (van Olst et al., 2022). Notably, two necroptosis clusters of AD samples that were identified through consensus clustering displayed unique innate immunological milieux, especially for the NK cells and macrophages. *In vitro* studies have demonstrated the functions of macrophages in the brain parenchyma, *viz.*, microglia, which promote early synapse loss in AD. Secreted phosphoprotein 1 (SPP1) produced predominantly by perivascular macrophages has been shown to drive microglial phagocytosis of synapses, thereby contributing to the development of amyloid-induced neurodegeneration (De Schepper et al., 2023). Likewise, NK cells were found to contribute to neuroinflammation and AD-associated cognitive decline (Zhang et al., 2020), suggesting that targeting the innate immune cells might be novel avenues for the treatment of AD (Dubois et al., 2023). Such observations highlight a sophisticated crosstalk orchestrated by varies cellular identities in the microenvironment driven by innate and adaptive immunity, which provide a mechanistic link between pathologic neuroimmune responses and disease phenotypes of AD.

Lately, machine learning-based algorithms, combing the clinical, digital pathology, multi-omics data, have been developed and applied in the diagnosis of diseases (Gao et al., 2022), drug developments (Rodriguez et al., 2021), and explorations of AD mechanisms (Tandon et al., 2023). Such algorithms provide valuable tools for interpreting the heterogeneity and classifications of diseases. A large body of evidence suggests that machine learning-based algorithms yield higher accuracies and efficiencies in the diagnosis of AD, outperforming clinical conventional approaches, e.g., traditional physical examinations, neuropsychological testing, and magnetic resonance imaging (MRI; Qiu et al., 2022). However, previous studies on such area have been restricted to a single learning algorithm, and the robustness of previously constructed diagnostic models has been hampered by the small sample sizes and limited validation cohorts. In the current study, we constructed a diagnostic model for AD using four machine learning-based algorithms. Among them, XGB presented the most outstanding stability and accuracy compared with other machine learning algorithms, and the five most important variables, including ACAA2, BHLHB4, CACNA2D3, NRN1, and TAC1, were used as hub genes for further analysis. Subsequently, two independent validation datasets were developed to evaluate the predictive potential of the five-gene diagnostic model, and results showed that the constructed model achieved excellent discrimination performance and stability, suggesting that it may be valuable in clinical settings. This also illustrated the significance of such hub genes in the development of AD.

The CACNA2D3 gene encodes the α-2δ subunit of the voltage-dependent calcium ion channel, and is known to contribute to late-onset AD following genomic imbalance (Villela et al., 2016). Besides, TAC1 encodes preprotachykinin-1 and is convergently enriched in central nervous system. Its aberrant expression in AD patients and APP/PS1 mice has been documented in previous studies (Liu et al., 2021). Elsewhere, it was identified as a hub gene in patients with vascular dementia (Shu et al., 2022). There is evidence that NRN1 can exert neurotrophic effects to participate in synaptic maintenance and neuronal survival (Sato et al., 2012), with some data suggesting that it may be closely associated with synaptic maturation, long-lasting stability, and activity-related plasticity (Picard et al., 2014; Subramanian et al., 2019). Of particular interest, a recent study by Hurst et al., demonstrated the underlying connections of NRN1 with the cognitive resilience in AD, and revealed its key role in facilitating dendritic spine resilience against Aβ in cultured neurons (Hurst et al., 2023). Notably, we observed that NRN1 and TAC1 were influenced by Aβ 42, β-, and γ-secretase activities, demonstrating that they are potential indicators for assessing the severity of pathologic alterations of AD. This further highlighted that such genes may influence the pathological phenotypes of AD. Besides the above genes, the ACAA2 gene encodes an enzyme of the thiolase family, which is an essential factor involved in β oxidation of lipid acids (Lei et al., 2022), while BHLHB4 is a transcription factor that regulates rod bipolar cell maturation (Bramblett et al., 2004). Currently, no study has explored the association of such two genes with AD.

Despite the important findings of this study, there are several limitations that need to be considered. First, it should be acknowledged that the disease cohorts investigated in this study were primarily derived from public database. Therefore, the reliability of the diagnostic models should be validated using external datasets. Further, *in vivo* and *in vitro* data are needed to elucidate the detailed mechanism of the identified hub genes in the pathophysiology of AD.

## Conclusion

Collectively, our study presents the expression profiles of NRGs in AD and normal individuals, and further reveals the distinct necroptosis clusters identified using consensus clustering in diseased samples, each with unique immune profiles. Moreover, a five-gene XGB-based diagnostic model for AD was constructed, which performed well in the training and independent validation datasets. Our work provides a novel diagnostic model that may serve as a framework to study disease heterogeneity and the mechanism underlying neuronal loss in AD.

## Data availability statement

The original contributions presented in the study are included in the article/Supplementary material, further inquiries can be directed to the corresponding authors.

## Author contributions

LL designed the study, drafted the manuscript, provided the funding, and supervised the study. HW, CW, and YY performed literature search, conducted statistical analyses, and visualized the results. All authors contributed to the article and approved the submitted version.

## Footnotes

^1^ https://www.ncbi.nlm.nih.gov/geo/

^2^ http://deathbase.org/

^3^ http://www.gsea-msigdb.org/gsea/index.jsp

^4^ https://www.genecards.org/

^5^ https://www.gsea-msigdb.org/gsea/msigdb

## Supplementary material

Supplementary table S1 Specific gene lists for intersectional analysis of the key genes obtained in WGCNA

Supplementary Figure 1 The immune landscape of AD samples of the two necroptosis clusters determined using the CIBERSORT algorithm. **(A)** Heatmap showing the relative proportions of infiltrating immune cells of two necroptosis clusters. **(B)** Barplot showing the differential analysis of various infiltrating immune cells between two necroptosis clusters. **p* < 0.05, ***p* < 0.01.

Supplementary Figure 2 Clinical correlation analysis for the hub genes included in the diagnostic model. **(A)** Association of CACNA2D3 with β-secretase activity. **(B-C)** Association of ACAA2 and NRN1 with age.
