# Supplementary figures and images for: Uncovering the Achilles heel of genetic heterogeneity: machine learning-based classification and immunological properties of necroptosis clusters in Alzheimer’s disease
Source: Front Aging Neurosci. 2023 Sep 20;15:1249682. doi: 10.3389/fnagi.2023.1249682 (PMC10548137; doi:10.3389/fnagi.2023.1249682)

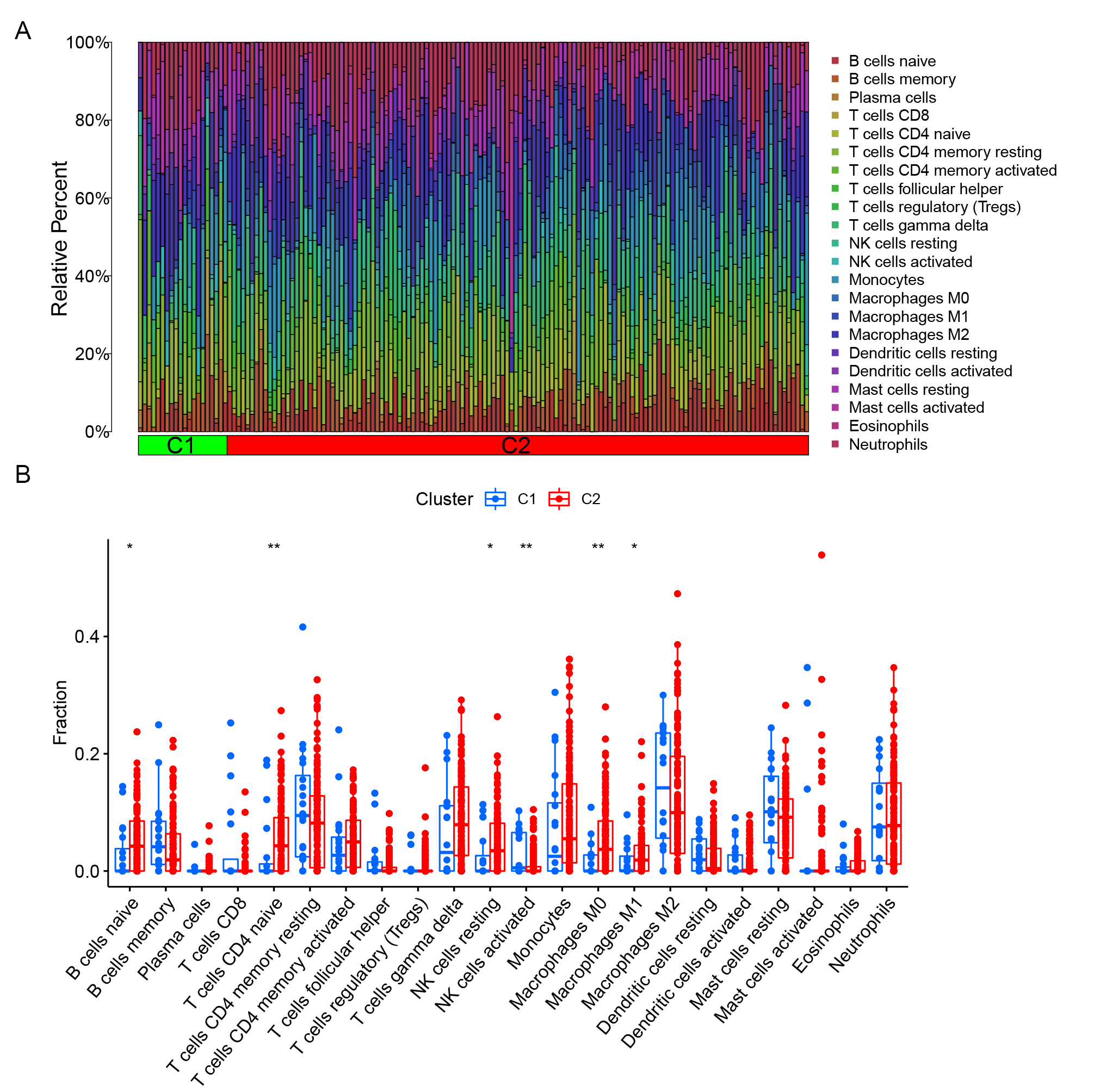

Supplement: Supplementary Figure 1 — The immune landscape of AD samples of the two necroptosis clusters determined using the CIBERSORT algorithm. (A) Heatmap showing the relative proportions of infiltrating immune cells of two necroptosis clusters. (B) Barplot showing the differential analysis of various infiltrating immune cells between two necroptosis clusters. *p < 0.05, **p < 0.01. [file Image_1.TIF]

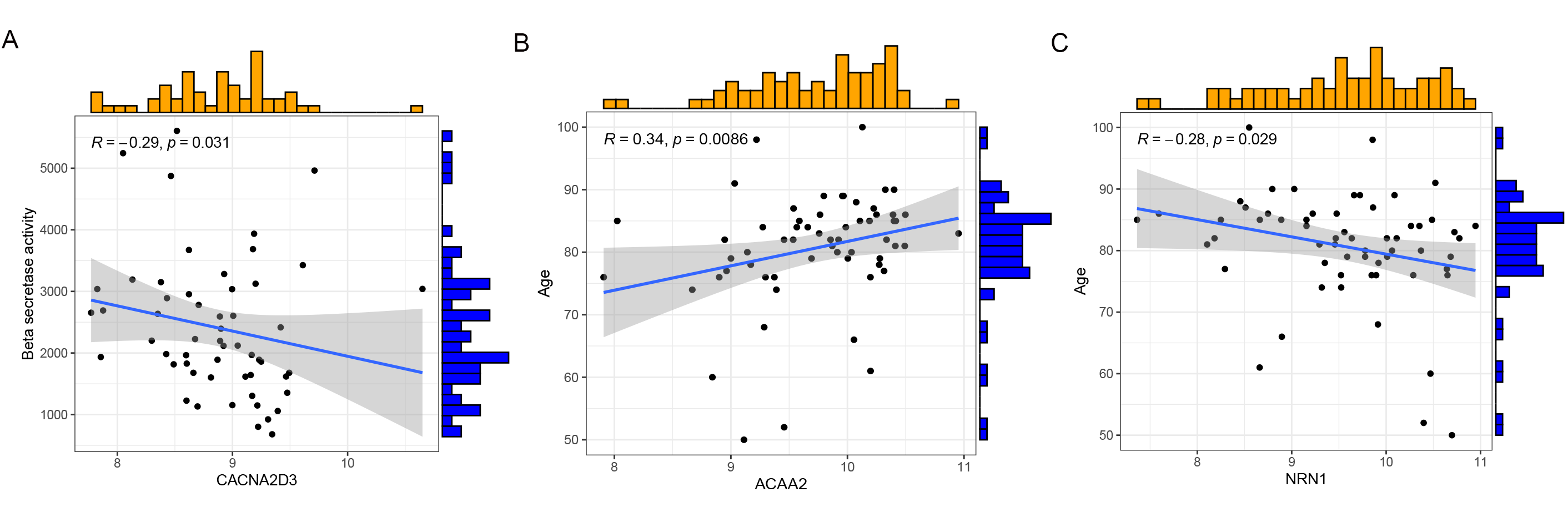

Supplement: Supplementary Figure 2 — Clinical correlation analysis for the hub genes included in the diagnostic model. (A) Association of CACNA2D3 with β-secretase activity. (B-C) Association of ACAA2 and NRN1 with age. [file Image_2.TIF]
